# Hospital Meetings

**Published:** 1904-04-02

**Authors:** 


### HOSPITAL MEETINGS.

### MOUNT VERNON HOSPITAL.

The annual meeting of the Mount Vernon Hospital for Consumption and Diseases of the Chest, Hampstead and Northwood, took place on Wednesday, March 23rd, at the Offices, 7 Fitzroy Square, W. Mr. J. S. Fletcher, Vice-President, occupied the chair.

The Chairman, in moving the adoption of the report and accounts for 1903, referred to the loss sustained by the Hospital through the death, on February 8th, of Mr. Benjamin A. Lyon, Chairman of the Committee of Management. Upon Mr. Lyon, who was first appointed to the chairmanship nearly 20 years ago, at a time of great stress and difficulty, had fallen the heavy burden of directing the policy of the hospital, and of organising its resources; and to him the interests of this institution were paramount and never far from his thoughts. His energy, alertness, and generosity were qualities which contributed in a high degree to the present prosperity of the hospital; but perhaps his most distinguishing characteristic was the power, rare in an Englishman, of stimulating generosity in others. Many Englishmen, Mr. Fletcher continued, were liable to fail in the task of raising funds for an institution through the nervous way in which they set about their task. Mr. Lyon, a generous giver himself, had the gifts that made work of this kind successful; his generosity was contagious, and he had his reward in seeing this hospital one of the most prosperous in Europe. The Committee and Governors desired most respectfully to tender their deepest sympathy to the relatives of their late Chairman, in whom they had lost a warm-hearted friend and colleague. Nor had this been the only loss. After the report was written the Committee had learnt with deep regret of the decease of Miss Flora Goldsmid, for many years associated with them in their work; her loss was a heavy one, and created a void which it would be difficult to fill. By the death of Sir John Blundell Maple, also, the hospital had lost a generous benefactor.

Turning to the report, the Chairman said the record for 1903 showed progress in almost every respect. The number of in-patients was 560, an increase of 13 on that of the previous year; while the daily average number of new with the old building was in progress the average residence was 61.3, compared with 61.4 days in 1902. During a portion of the year, while the joining up of the new with the old building was in progress the average number of beds available was reduced to 90. On the opening of the new block in October, however, the number was increased to 145; but it was not proposed to open more than 15 extra beds until the debt on the extensions—£15,000—had been paid off. The number of out-patients had also increased, being 5,762, against 4,962 in the previous year.

During the last ten years the work of the Hospital had more than doubled, and were there sufficient funds to maintain all the beds at least 100 additional patients could be admitted annually, thereby greatly reducing the long list of waiting applicants.

The Committee were gratified to be enabled to report an increase in annual subscriptions and donations; the total income, however, must be still further augumented to meet increasing claims. The increase already accomplished was largely due to the efforts put forth in connection with the Festival Dinner held on May 20th last, under the Presidency of Viscount Knutsford, G.C.M.G., at which more than £2,000 was raised. This was the second occasion on which Viscount Knutsford had rendered this service to the Hospital, and the Committee desired to place on record their sincere thanks for his valuable co-operation. Their thanks were due also to the representatives of King Edward's Hospital Fund for the continuance of their annual subscription of £1,000, and a donation of £500; also to the Sunday and Saturday Funds for increased grants, £780 and £457 18s. respectively; and to the Bishop of Kensington for his advocacy of the claims of the Hospital at Hampstead Parish Church in October last. A comparison of the expenditure with that of previous years showed that the Hospital was conducted on economical principles. The House Committee exercised very careful supervision over all domestic expenditure, and the result was reflected in the accounts. While the cost of administration had been kept within reasonable limits, and compared very favourably with that of former years, the extraordinary expenditure had been heavy. The various alterations consequent upon the completion of the building, the acquisition and repair of Mount Vernon House for the nurses, and the cost of furnishing this and the new block of the hospital, had involved considerable expense, while the same observation applied to the completion of the out-patients' department in Fitzroy Square. The Committee regretted to have to report a deficiency of £11,000 on the Building Fund Account. This sum had been advanced by way of loan, and the Committee very earnestly pleaded for help to enable them to repay this amount, and to meet outstanding charges, amounting to about £4,000, on this account. The Committee had much satisfaction in reporting that the new sanatorium at Northwood was approaching completion, and would, it was hoped, be ready for occupation in June. After much consideration and consultation with the Hon. Medical Staff they had appointed Dr. T. N. Kelynack to be Hon. Physician in Charge, to reside at Northwood, and to represent his colleagues in the work of the sanatorium. Miss Florence Swain, until lately a sister in the hospital, had been elected to fill the post of Matron; and the Rev. E. Batt Backhouse, Vicar of Northwood, had been appointed Chaplain.

The motion, having been seconded by Col. Arthur, was carried. The retiring members of the Committee were then re-elected; Dr. J. Fletcher Little, retiring member of the Hon. Medical Staff, was elected an Hon. Governor of the Hospital, and the by-laws were amended so as to be applicable to the Sanatorium at Northwood as well as to the Hospital at Hampstead, provision being made for a House Committee at Northwood.

A vote of thanks to the Chairman, proposed by Mr. Malcolm, seconded by Col. Arthur and supported by Mr. F. D. Mocatta, who said he hoped the time would come when all Chest Hospitals were able to keep their patients as long as they were receiving benefit from the treatment, was carried unanimously.

### ST. MARY'S HOSPITAL.

The annual meeting of St. Mary's Hospital was held on Thursday, March 24th; Colonel Bannerman in the Chair.

The adoption of the report and accounts for 1903, together with votes of thanks to the various officers and to the medical, surgical, and nursing staffs, was put from the Chair. The Board of Management reported that the number of out-patients was greater than in the previous year by 255, and that of the in-patients by 96. The electric-light installation in the old hospital was now nearly completed, and the wiring of the Clarence wing was in progress. By its means, with a light between every two beds, the patients were able to read with comfort; while by means of wall plugs and hand lamps, a good light was immediately available in any position, an advantage of the utmost value to the staff. The estimate for this work was £3,200, and the payments up to December 31 were £1,249. This item largely accounted for the heavy extraordinary expenditure, amounting to £3,442, the remainder being disbursed for building improvements. With regard to ordinary expenditure, the only class in which an increase was not shown was that of provisions, which had cost less by £244 than in 1902. The total ordinary expenditure exceeded that of the previous year by £1,646. Of the items included, the largest was that of the nurses' salaries. It was important that it should be known that the demands on the nursing staff had become much heavier of late years, mainly because of the great increase in the number and importance of surgical operations, and that now it would be quite impossible for the work to be efficiently carried on with the staff of five years ago. In 1903 six nurses were added to the staff, and with more accommodation the number would have been larger still, while every year additions became necessary. The number of supernumerary nurses engaged for special work in times of pressure also tended constantly to increase.

With regard to the Clarence Memorial wing, the building works had continued throughout the year, and the main part of the structure was now completed and roofed in. There was every reason to hope that the remainder would be finished by the date fixed in the building contract —viz. January, 1905. The funds necessary to complete and furnish this wing were not, however, in hand; and as labour and materials had increased in cost during the long time the building had been in progress, it was probable that the additional sum required to supplement the Zung bequest would considerably exceed the figure at which it had been put—viz. £10,000. The Board had much satisfaction in announcing that during the year negotiations, which had been in progress for three years, for acquiring the freehold of certain parts of the hospital site had been brought to a successful issue, and the whole of the site was now freehold.

The total income from all sources during 1903 was less by £9,575, the explanation being that in 1902 legacies and donations for the endowment of beds reached the high total of £11,367, and that there was also a special donation of £3,300 for the benefit of the medical school, making a total for this class of income of £14,667, while the corresponding figure for 1903 was £3,250, a decrease of £11,417. Notwithstanding this falling-off in the total, however, the Board recognised with pleasure that the amount given for the purpose of the endowment of beds showed a gratifying improvement, and that the income available to meet the expenditure of the year was £2,091 more than in 1902. This improvement would have been more satisfactory had they been able to classify it as ordinary income. Unfortunately, the improvement was confined to legacies, which amounted to £14,399, while the ordinary income was £14,810, being less, by £39, than that of the previous year.

In view of the approaching completion of the Clarence wing, and the consequent increase in the work, and therefore in the expenditure of the hospital, the Board felt that it was of the utmost importance to look ahead and seriously consider the financial position. It must be remembered that the enlargement of the building had been rendered necessary by the great increase in the work the charity was called on to perform. This increase was largely due to the great growth in the population of the district served by the hospital—a district in which, perhaps, more than in any other (to quote the late Mr. Gladstone), "boundless wealth and boundless want subsisted side by side." It had been found impossible with the present accommodation to provide efficiently for the treatment of the ever-increasing numbers applying for relief, and the enlargement and improvement of equipment were therefore works of necessity. In this connection it might be mentioned that the system of inquiry into the circumstances of out-patients, established in 1898, with the view of preventing abuse of the charity by those able to pay for treatment, had been continued throughout 1903.

The scheme of improvements, including the new wing, new boilers and boiler-house, and laundry equipment, required a sum of £25,000; and until these items were completed the hospital could not be effectively worked. The most pressing financial feature of all, however, was the necessary addition to the annual income. The number of beds would be increased by about 60, the average annual cost of which, at the present rate of expenditure—£100 a bed—would represent a sum of £6,000 a year. As, however, some classes of expenditure would not be affected by the opening of the new wing, and some would be actually reduced, the annual charge might be estimated at £5,000. The task before the Board—viz. the raising of an additional annual income of £5,000, and of an immediate sum of £25,000, was a formidable one; but it must be faced, and it would be strenuously and perseveringly taken in hand. The Board now appealed for help to the public, and especially the residents in the immediate neighbourhood of Paddington, Kensington, and Marylebone, and particularly the last two, for which the hospital did so much, and from which it received so little. In recent reports to the Borough Council, the medical officer of Kensington had dwelt on the subject of the extent to which that wealthy borough depended for the relief of its sick poor on St. Mary's Hospital, which, in 1903, spent no less a sum than £4,500 in the relief of Kensington patients alone. The facts were almost the same with regard to Marylebone. The Board, therefore, felt justified in appealing to the wealthy residents of these boroughs, and especially those of the Royal borough, to assist them in their efforts to provide adequately for the district served by the hospital, and to keep pace with the rapid advance of the science of healing and nursing. The hospital had been opened but 53 years, and its financial foundations were, in consequence, all too narrow, and its investments, the result of careful economy in the past, produced only £2,000 a year, while the expenditure must be put down as something like £30,000.

Mr. Smith having seconded the motion, it was unanimously adopted, and the meeting adjourned.

---

### THE ROYAL NATIONAL HOSPITAL FOR CONSUMPTION, VENTNOR.

The annual meeting of the Royal National Hospital for Consumption, Ventnor, took place on March 24, at the offices, 34 Craven Street, Charing Cross.

The Chairman of the Board (Mr. P. B. Burgoyne) presided.

In moving the adoption of the report and statement of accounts, he said that, before dealing with the business on the agenda, he desired to refer to the great loss the hospital had sustained through the death of an illustrious Patron and Governor, the Duke of Cambridge, who had most graciously associated himself with the work of the hospital. He presided at the hospital Dinner in 1887, and in various ways was instrumental in adding materially to the funds. His death was the severance of an important link with the good deeds of the past, in which Her Majesty Queen Victoria, of blessed memory, was always foremost. At a meeting of the Board to be held afterwards, a vote of condolence with Colonel FitzGeorge would be moved. The accounts showed a deficiency of £791, and he wished to make this pronounced. For one thing, a larger number of patients had been treated; and, for another, the patients had been better fed, the meat bill for the last year being £2,123, as against £1,707 in the previous year. Funds were urgently needed. He did not consider that people were less charitable now than they used to be. It was simply that the channels were diverted; they were not dried up. The hospital did not share in King Edward's Hospital Fund because of a rule that the Fund should be allotted to institutions in the County of London. But they hoped that the Council of the Fund would recognise the benefits conferred on poor consumptives by this hospital, and vote it a grant, seeing that nearly half the patients were from the Metropolis. The report showed the increasingly good work the hospital was doing. During the past year there were 795 completed cases, and of these 417 left much improved; 261 left improved; 78 remained practically unchanged; 18 became worse; and 21 died. The number who gained weight was 708; of those who lost weight, 61. There were 26 fatal and unweighed cases; but the average gain was 9 lb. 4.7 oz. The plan adopted by the Governors last year with regard to the method of issuing letters of recommendation has answered admirably, and, instead of 270 applicants waiting for admission, the number had been reduced to 70. He was happy to state that throughout the county there had been an increase of 375 beds for consumptives, but the total number of beds—2,760—fell far short of the requirements. There had been an increase of deaths from consumption during the past year.

Mr. Powell seconded the motion, and said that they were now adopting the open-air treatment as far as possible in place of the artificial ventilation, which had proved a failure. Ventilation, he considered, was a science of the highest importance.

Dr. Robertson, supporting the motion, spoke encouragingly of the uninterrupted improvements and said that no less than three-fourths of the patients had been relieved from their sufferings. The disease, he said, was a tedious one, and it was most necessary that it should be treated in its early stages, which, unfortunately, was seldom the case with people who were too poor to give up their work till they were compelled to do so. This institution, he pointed out, was not a sanatorium, but a hospital for the benefit of those necessitous people who, otherwise, would be obliged to suffer without relief.

The resolution was carried.

Lord Alverstone, in proposing a vote of thanks to the Chairman, said that only those on the Board could realise the constant attention and sacrifice of time which Mr. Burgoyne gave to the interests of the hospital.

The unanimous carrying of this motion brought the meeting to a close.

### BRITISH LYING-IN HOSPITAL.

The annual general meeting of the British Lying-in Hospital, Endell Street, W.C., was held in the afternoon of Thursday, March 24th, Mr. Frederick Cox in the Chair.

The Chairman, in rising to move the adoption of the report and balance sheet for 1903, said he would call on Mr. Charles E. Farmer, the Chairman of the Board of Management, to place before the meeting his views as to the position and needs of the charity. At the same time he would like to say what a keen interest he himself had taken in the hospital's affairs in the past, and he might perhaps add that he had been connected with it for the past 35 years. He had that afternoon inspected the new Nurses' Home, wards, covered way, and side entrance for patients, and could only say that he was immensely pleased with what he had seen. The alterations would vastly improve the working of all departments and the comfort of the pupils and nurses, but they would require additional funds for their upkeep.

Mr. Farmer, in supporting the motion, said that the report showed that the past year had been a memorable one in the annals of the institution, as it had witnessed the opening of the new Nurses' Home by H.R.H. the Princess of Wales, and the addition of two more wards. Her Royal Highness had graciously extended her patronage to the hospital, and under such patronage, and with its extended sphere of usefulness, the institution could not fail to be of largely increased benefit to those in whose interest it had for more than 150 years been working. The alterations in the building, while in progress, interfered to some extent with the working of the hospital, and the new wards did not come into use until late in the autumn, so that the number of in-patients does not show the increase which may reasonably be expected when the re-arrangements have had a year's trial. The work now in progress largely exceeds that of former years. The money expended on the Home and two new wards amounted to £7,178, of which more than £5,000 had to be raised out of the invested funds, towards the repayment of which further donations are most earnestly solicited.

The number of in-patients in 1903 was 438, being an increase of 23 on 1902; and the out-patients 732, an increase of 57 on 1902. There were no maternal deaths. In consequence of the growth of work an additional medical officer to out-patients had become necessary, and Dr. J. S. Fairbairn had been appointed to the post.

The hospital had been approved by the Council of the Central Midwives' Board as a training school for midwives, and its certificate is now accepted by the Board as sufficient qualification for a certificated midwife.

The hospital was indebted to Dr. John Phillips for the addition of a covered-way side entrance for patients, which he had kindly provided for out of a sum of money at his disposal for charitable purposes, together with a small income in order to keep it in repair.

The Kyrle Society had kindly presented 24 pictures for the Home sitting and dining room, and were going to undertake the stencilling of a ward.

### GORDON HOSPITAL FOR FISTULA AND OTHER DISEASES OF THE RECTUM.

The annual meeting of the Governors of this Hospital was held on March 24. The chair was taken by Mr. Arthur Hughes (Chairman of the General Committee), who, in moving the adoption of the report, remarked that during the past year they had admitted 35 more in-patients to the hospital than in 1902. The number of out-patients had remained the same. The medical staff of the hospital and the committee had long been desirous of having a resident house surgeon, and through the exertions of Mr. Cecil Leaf, a member of the surgical staff, who raised a sum of £89, they had been enabled to appoint one. This step had placed the hospital on a proper basis, and relieved the matron of much anxiety. With regard to the hospital accounts, they were not quite so satisfactory as they looked, for the balance in hand of £116 contained the sum of £89 collected for the salary of the house surgeon. Also, it so happened that the profits from two sets of their annual dances were included in this year's accounts, but in future they would only include the profits of one set. The hospital was burdened with a liability of £6,950 under a covenant in a lease from the Ecclesiastical Commissioners, and unless they were enabled to find this amount to complete building by the end of the year they would be compelled to abandon the lease. They also required £175 additional income in order to pay their way. The report showed that grants had been received from King Edward's Hospital Fund and from the Hospital Sunday Fund.

